# An inter­molecular dative B←N bond in 5-(4,4,5,5-tetra­methyl-1,3,2-dioxa­borolan-2-yl)-1,3-thia­zole

**DOI:** 10.1107/S1600536809052775

**Published:** 2009-12-16

**Authors:** Jana Sopková-de Oliveira Santos, Nicolas Primas, Jean-François Lohier, Alexandre Bouillon, Sylvain Rault

**Affiliations:** a Centre d’Études et de Recherche sur le Médicament de Normandie (CERMN), UPRES EA-4258, FR CNRS INC3M, Université de Caen, bv becquerel, 14032 Caen, France; b Laboratoire de Chimie Moléculaire et Thio-organique, UMR CNRS 6507, UPRES EA-4258, FR CNRS 3038 INC3M, ENSICAEN - Université de Caen, 14050 Caen, France; c BoroChem S.A.S., Immeuble Emergence, 7 rue Alfred Kastler, 14000 Caen, France

## Abstract

The title compound, C_9_H_14_BNO_2_S, is in an unusual bend conformation and the B atom of one mol­ecule within the crystal forms an inter­molecular dative bond with the N atom of a neighbouring mol­ecule, an infrequent phenomenon in boronic derivative crystals.

## Related literature

For related natural compounds, see: Dondoni & Merino (1996 ▶); Faulkner (1998 ▶); Hutchinson *et al.* (2000 ▶); Kalgutkar *et al.* (1996 ▶); Ogino *et al.* (1996 ▶); Williams & Jacobs (1993 ▶). For boronic esters, see: Allen (2002 ▶); Höpfl (1999 ▶); Hall (2005 ▶); Rettig & Trotter (1975 ▶); Sopková-de Oliveira Santos *et al.* (2003*a* ▶,*b* ▶). For details of the synthesis, see: Primas *et al.* (2008 ▶, 2009 ▶).
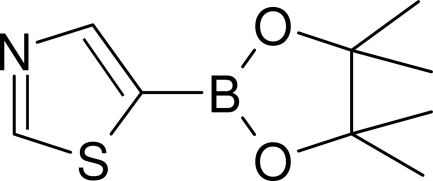


## Experimental

### 

#### Crystal data

C_9_H_14_BNO_2_S
*M* *_r_* = 211.08
Monoclinic, 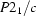

*a* = 12.6169 (3) Å
*b* = 7.9845 (2) Å
*c* = 12.6679 (3) Å
β = 119.064 (1)°
*V* = 1115.46 (5) Å^3^
*Z* = 4
Mo *K*α radiation
μ = 0.26 mm^−1^
*T* = 296 K
0.53 × 0.36 × 0.32 mm

#### Data collection

Bruker APEXII CCD area-detector diffractometer
42058 measured reflections
5399 independent reflections
3895 reflections with *I* > 2σ(*I*)
*R*_int_ = 0.023

#### Refinement

*R*[*F* ^2^ > 2σ(*F* ^2^)] = 0.046
*wR*(*F* ^2^) = 0.159
*S* = 1.04
5399 reflections
139 parameters
H atoms treated by a mixture of independent and constrained refinement
Δρ_max_ = 0.87 e Å^−3^
Δρ_min_ = −0.68 e Å^−3^

### 

Data collection: *APEX2* (Bruker, 2006 ▶); cell refinement: *SAINT* (Bruker, 2006 ▶); data reduction: *SAINT*; program(s) used to solve structure: *SHELXS97* (Sheldrick, 2008 ▶); program(s) used to refine structure: *SHELXL97* (Sheldrick, 2008 ▶); molecular graphics: *ORTEP-3* (Farrugia, 1997 ▶) and *PLATON* (Spek, 2009 ▶); software used to prepare material for publication: *SHELXL97*.

## Supplementary Material

Crystal structure: contains datablocks I, global. DOI: 10.1107/S1600536809052775/dn2507sup1.cif

Structure factors: contains datablocks I. DOI: 10.1107/S1600536809052775/dn2507Isup2.hkl

Additional supplementary materials:  crystallographic information; 3D view; checkCIF report

## Figures and Tables

**Table 1:** Hydrogen-bond geometry (Å, °)

*D*—H⋯*A*	*D*—H	H⋯*A*	*D*⋯*A*	*D*—H⋯*A*
C2—H2⋯O1^i^	0.94 (2)	2.36 (2)	3.2446 (14)	157.5 (17)

Symmetry code: (i) 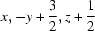.

**Fig. 1.**
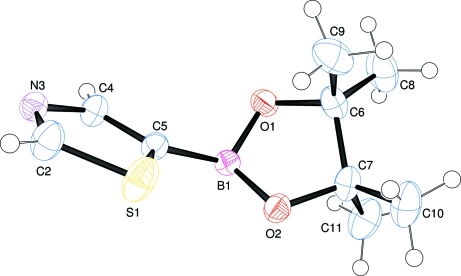
View of the title compound showing the labelling scheme of the non-hydrogen atoms. Thermal ellipsoids are shown at the 50% probability levels; hydrogen atoms are drawn as small circles of arbitrary radii.

**Fig. 2.**
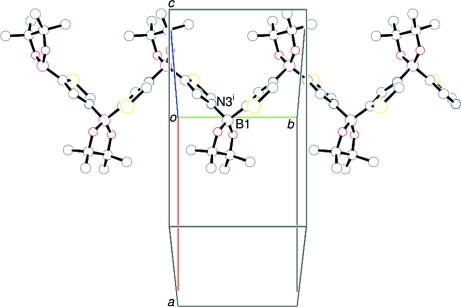
Partial packing view showing the chain formed by intermolecular dative B←N bonds. [Symmetry code: (i) -x, y - 1/2, -z + 1/2)]

**Fig. 3.**
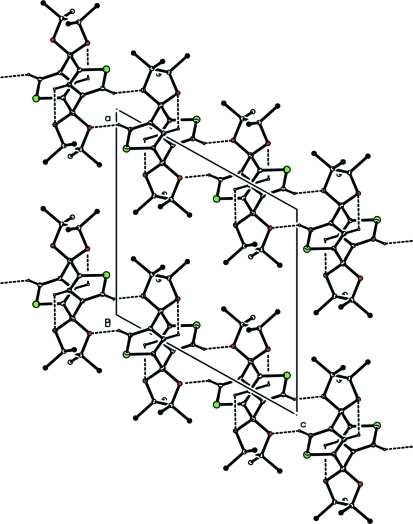
Packing view showing the C-H···O hydrogen bonds connecting the chain. H atoms not involved in hydrogen bondings have been omitted for clarity. H bonds are shown as dashed lines.

## supplementary crystallographic information

### Comment

The thiazole ring is a widespread heterocycle found in various biologically active natural products like vitamin B1 and penicillins (Dondoni & Merino, 1996; Kalgutkar *et al.*, 1996; Hutchinson *et al.*, 2000), and also in many peptides and peptolides isolated from marine organisms (Ogino *et al.*, 1996; Williams & Jacobs, 1993; Faulkner, 1998). The search of new regioselective methods for the preparation of new thiazole derivatives is always a matter of interest.

As a part of our study of 1,3-azolylboronic derivatives, we are focused on the synthesis of the new thiazol-5-ylboronic acid pinacol ester (Primas *et al.*, 2009). Indeed, this new boronic ester permits a facile synthetic route to 5-(het)arylthiazoles *via* a Suzuki-Miyaura cross-coupling reaction with various (het)halides. This boronic ester was stable under aqueous conditions in the Suzuki process (Primas *et al.*, 2008). To date only a few crystallographic studies have been published on such heterocyclic compounds. We carried out this study with the aim to confirm the structure of the title compound and to find an explanation to its greater stability than its imidazole analogue (Primas *et al.*, 2008).

The structure shows that the molecule is in an unusual bent conformation, thus the thiazole cycle and the dioxaborolane ring of the boronic ester forms an angle of about 55.0(0.05)° (Fig. 1). Usually, in the boronic esters deposed in Cambridge Structural Database (CSD, Version; Allen 2002) as well as in the ones solved previously in our laboratory, the ester ring is coplanar to the aromatic ring (Sopková-de Oliveira Santos *et al.*, 2003*a*,b).

In the crystal structure the boron atom is the peak bending, and it is committed to the B←N dative bond with N of the neighbouring molecule (-*x*,*y* - 1/2, -*z* + 1/2), which leads to a tetracoordinated B atom in the crystal. As it was already published (Hall, 2005), the formation of tetracoordinate B influences all bond lenghts in the boron vicinity. The observed B—O bond lengths, 1.4351 (13)Å and 1.4447 (13) Å, are in agreement with the ones reported when B is tetracoordinated (Hall, 2005), between 1.43–1.47 Å. The observed C—B distance is about 1.6207 (15)Å which is closed to the usually observed value for tetracoordinate boron, 1.613Å (Rettig & Trotter, 1975). However, the B←N dative bond observed in the crystal is shorter with respect to the published value, its length is about 1.6354 (14) Å. Furthermore, the calculated parameter describing the tetrahedral character of boron (THC_DA_; Höpfl, 1999) is in the title compound of 81% which is a high value and shows that the formed B←N dative bond is a strong one. The existence of this strong B←N dative bond could explain the stability of this boronic ester even not only in the solid state but also in the solution. Further studies concerning this phenomenon are currently in progress.

The dioxaborolane ring of the boronic ester is in a half-chair conformation with an O1—C6—C7—O2 torsion angle of about -37.74(0.10)°.

The crucial element of the crystal packing is of course this intermolecular dative B←N bond. The interacting neighbouring molecules form a strand along the *b* axis (Fig. 2). Some electrostatic interactions occur between these strands, the strongest seems to be an electrostatic interaction between O1 and H2—C2 of symmetrically related molecule (Table 2, Fig. 3).

### Experimental

The title compound was synthesized from 2-trimethylsilylthiazole using the method described by Primas *et al.* (2009). Crystals of (I) suitable for X-ray analysis were obtained by slow evaporation from diethyl ether at room temperature.

### Refinement

All non-hydrogen atoms were refined anisotropically. All H atoms were determined *via* difference Fourier map and refined with isotropic atomic displacement parameters with exception on H atoms on methyl groups which were calculated and fixed on the atoms in the ideal geometry (distance 0.96 Å).

### Crystal data

**Table:**

C_9_H_14_BNO_2_S	*F*(000) = 448
*M**_r_* = 211.08	*D*_x_ = 1.257 Mg m^−^^3^
Monoclinic, *P*2_1_/*c*	Melting point: 371 K
Hall symbol: -P 2ybc	Mo *K*α radiation, λ = 0.71073 Å
*a* = 12.6169 (3) Å	Cell parameters from 9543 reflections
*b* = 7.9845 (2) Å	θ = 3.2–35.8°
*c* = 12.6679 (3) Å	µ = 0.26 mm^−^^1^
β = 119.064 (1)°	*T* = 296 K
*V* = 1115.46 (5) Å^3^	Plate, colourless
*Z* = 4	0.53 × 0.36 × 0.32 mm

### Data collection

**Table:**

Bruker APEXII CCD area-detector diffractometer	3895 reflections with *I* > 2σ(*I*)
Radiation source: fine-focus sealed tube	*R*_int_ = 0.023
graphite	θ_max_ = 36.4°, θ_min_ = 1.9°
φ and ω scans	*h* = −21→21
42058 measured reflections	*k* = −10→13
5399 independent reflections	*l* = −21→21

### Refinement

**Table:**

Refinement on *F*^2^	Primary atom site location: structure-invariant direct methods
Least-squares matrix: full	Secondary atom site location: difference Fourier map
*R*[*F*^2^ > 2σ(*F*^2^)] = 0.046	Hydrogen site location: inferred from neighbouring sites
*wR*(*F*^2^) = 0.159	H atoms treated by a mixture of independent and constrained refinement
*S* = 1.04	*w* = 1/[σ^2^(*F*_o_^2^) + (0.0814*P*)^2^ + 0.2917*P*] where *P* = (*F*_o_^2^ + 2*F*_c_^2^)/3
5399 reflections	(Δ/σ)_max_ < 0.001
139 parameters	Δρ_max_ = 0.87 e Å^−^^3^
0 restraints	Δρ_min_ = −0.68 e Å^−^^3^

### Special details

**Table:**

Geometry. All e.s.d.'s (except the e.s.d. in the dihedral angle between two l.s. planes) are estimated using the full covariance matrix. The cell e.s.d.'s are taken into account individually in the estimation of e.s.d.'s in distances, angles and torsion angles; correlations between e.s.d.'s in cell parameters are only used when they are defined by crystal symmetry. An approximate (isotropic) treatment of cell e.s.d.'s is used for estimating e.s.d.'s involving l.s. planes.
Refinement. Refinement of *F*^2^ against ALL reflections. The weighted *R*-factor *wR* and goodness of fit *S* are based on *F*^2^, conventional *R*-factors *R* are based on *F*, with *F* set to zero for negative *F*^2^. The threshold expression of *F*^2^ > σ(*F*^2^) is used only for calculating *R*-factors(gt) *etc*. and is not relevant to the choice of reflections for refinement. *R*-factors based on *F*^2^ are statistically about twice as large as those based on *F*, and *R*- factors based on ALL data will be even larger.

### Fractional atomic coordinates and isotropic or equivalent isotropic displacement parameters (Å^2^)

**Table:**

	*x*	*y*	*z*	*U*_iso_*/*U*_eq_	
S1	0.16573 (3)	0.62779 (5)	0.44438 (3)	0.04720 (12)	
C2	0.06243 (12)	0.77776 (17)	0.42356 (10)	0.0386 (3)	
H2	0.0698 (18)	0.848 (3)	0.4859 (19)	0.057 (5)*	
N3	−0.02480 (8)	0.78577 (10)	0.31137 (8)	0.02725 (16)	
C4	−0.00940 (10)	0.67047 (14)	0.23876 (10)	0.0305 (2)	
H4	−0.0704 (14)	0.667 (2)	0.1570 (15)	0.040 (4)*	
C5	0.08968 (9)	0.57082 (12)	0.29535 (9)	0.02743 (18)	
B1	0.13725 (10)	0.41927 (13)	0.24402 (10)	0.02591 (19)	
O2	0.24223 (7)	0.34057 (10)	0.33978 (7)	0.02967 (16)	
O1	0.16342 (7)	0.46160 (10)	0.14816 (7)	0.03061 (16)	
C6	0.29217 (10)	0.43640 (17)	0.19662 (10)	0.0343 (2)	
C8	0.31785 (16)	0.3921 (3)	0.09484 (14)	0.0580 (4)	
H8A	0.4032	0.3733	0.1271	0.087*	
H8B	0.2925	0.4827	0.0378	0.087*	
H8C	0.2741	0.2924	0.0551	0.087*	
C9	0.35543 (14)	0.6010 (2)	0.25586 (15)	0.0492 (3)	
H9A	0.3213	0.6906	0.1985	0.074*	
H9B	0.4405	0.5919	0.2821	0.074*	
H9C	0.3441	0.6238	0.3241	0.074*	
C7	0.31999 (10)	0.29495 (15)	0.29088 (10)	0.0332 (2)	
C10	0.44987 (12)	0.2890 (2)	0.39326 (14)	0.0501 (3)	
H10A	0.4674	0.3891	0.4408	0.075*	
H10B	0.5041	0.2807	0.3605	0.075*	
H10C	0.4602	0.1933	0.4433	0.075*	
C11	0.28385 (15)	0.12147 (19)	0.23354 (16)	0.0519 (4)	
H11A	0.2824	0.0443	0.2909	0.078*	
H11B	0.3416	0.0842	0.2099	0.078*	
H11C	0.2048	0.1269	0.1638	0.078*	

### Atomic displacement parameters (Å^2^)

**Table:**

	*U*^11^	*U*^22^	*U*^33^	*U*^12^	*U*^13^	*U*^23^
S1	0.04648 (19)	0.0520 (2)	0.02922 (15)	0.02133 (14)	0.00746 (12)	−0.00218 (12)
C2	0.0423 (6)	0.0395 (6)	0.0296 (5)	0.0097 (5)	0.0139 (4)	−0.0041 (4)
N3	0.0284 (4)	0.0240 (3)	0.0294 (4)	0.0009 (3)	0.0141 (3)	−0.0013 (3)
C4	0.0298 (4)	0.0294 (4)	0.0292 (4)	0.0042 (3)	0.0119 (4)	−0.0028 (3)
C5	0.0281 (4)	0.0246 (4)	0.0301 (4)	0.0011 (3)	0.0146 (3)	0.0003 (3)
B1	0.0258 (4)	0.0243 (4)	0.0283 (4)	0.0008 (3)	0.0136 (4)	0.0013 (3)
O2	0.0293 (3)	0.0315 (3)	0.0278 (3)	0.0057 (3)	0.0136 (3)	0.0029 (3)
O1	0.0275 (3)	0.0366 (4)	0.0288 (3)	−0.0002 (3)	0.0145 (3)	0.0041 (3)
C6	0.0288 (4)	0.0471 (6)	0.0304 (4)	−0.0017 (4)	0.0171 (4)	−0.0033 (4)
C8	0.0505 (8)	0.0937 (13)	0.0402 (7)	0.0083 (8)	0.0303 (6)	−0.0025 (7)
C9	0.0424 (7)	0.0543 (8)	0.0497 (7)	−0.0174 (6)	0.0214 (6)	−0.0024 (6)
C7	0.0279 (4)	0.0376 (5)	0.0312 (5)	0.0053 (4)	0.0120 (4)	−0.0054 (4)
C10	0.0312 (5)	0.0667 (9)	0.0426 (6)	0.0123 (6)	0.0103 (5)	−0.0041 (6)
C11	0.0541 (8)	0.0399 (6)	0.0550 (8)	0.0094 (6)	0.0212 (7)	−0.0123 (6)

### Geometric parameters (Å, °)

**Table:**

S1—C2	1.6936 (12)	C6—C7	1.5548 (17)
S1—C5	1.7122 (11)	C8—H8A	0.9600
C2—N3	1.3097 (15)	C8—H8B	0.9600
C2—H2	0.94 (2)	C8—H8C	0.9600
N3—C4	1.3803 (13)	C9—H9A	0.9600
N3—B1^i^	1.6354 (14)	C9—H9B	0.9600
C4—C5	1.3561 (14)	C9—H9C	0.9600
C4—H4	0.945 (16)	C7—C10	1.5182 (17)
C5—B1	1.6207 (15)	C7—C11	1.5273 (18)
B1—O2	1.4351 (13)	C10—H10A	0.9600
B1—O1	1.4447 (13)	C10—H10B	0.9600
B1—N3^ii^	1.6354 (14)	C10—H10C	0.9600
O2—C7	1.4386 (13)	C11—H11A	0.9600
O1—C6	1.4448 (13)	C11—H11B	0.9600
C6—C8	1.5159 (17)	C11—H11C	0.9600
C6—C9	1.5318 (19)		
			
C2—S1—C5	92.26 (5)	H8A—C8—H8B	109.5
N3—C2—S1	112.39 (8)	C6—C8—H8C	109.5
N3—C2—H2	125.0 (13)	H8A—C8—H8C	109.5
S1—C2—H2	122.6 (13)	H8B—C8—H8C	109.5
C2—N3—C4	111.99 (9)	C6—C9—H9A	109.5
C2—N3—B1^i^	126.44 (9)	C6—C9—H9B	109.5
C4—N3—B1^i^	121.50 (8)	H9A—C9—H9B	109.5
C5—C4—N3	115.54 (10)	C6—C9—H9C	109.5
C5—C4—H4	127.7 (11)	H9A—C9—H9C	109.5
N3—C4—H4	116.7 (11)	H9B—C9—H9C	109.5
C4—C5—B1	130.68 (9)	O2—C7—C10	108.61 (10)
C4—C5—S1	107.81 (8)	O2—C7—C11	109.02 (11)
B1—C5—S1	121.49 (7)	C10—C7—C11	109.07 (12)
O2—B1—O1	108.61 (8)	O2—C7—C6	101.54 (8)
O2—B1—C5	110.94 (8)	C10—C7—C6	115.23 (12)
O1—B1—C5	116.29 (8)	C11—C7—C6	112.94 (11)
O2—B1—N3^ii^	109.23 (8)	C7—C10—H10A	109.5
O1—B1—N3^ii^	107.26 (8)	C7—C10—H10B	109.5
C5—B1—N3^ii^	104.22 (8)	H10A—C10—H10B	109.5
B1—O2—C7	106.82 (8)	C7—C10—H10C	109.5
B1—O1—C6	106.21 (8)	H10A—C10—H10C	109.5
O1—C6—C8	109.38 (10)	H10B—C10—H10C	109.5
O1—C6—C9	107.41 (11)	C7—C11—H11A	109.5
C8—C6—C9	109.94 (12)	C7—C11—H11B	109.5
O1—C6—C7	102.43 (8)	H11A—C11—H11B	109.5
C8—C6—C7	114.97 (12)	C7—C11—H11C	109.5
C9—C6—C7	112.19 (10)	H11A—C11—H11C	109.5
C6—C8—H8A	109.5	H11B—C11—H11C	109.5
C6—C8—H8B	109.5		
			
O1—C6—C7—O2	−37.74 (10)		

Symmetry codes: (i) −*x*, *y*+1/2, −*z*+1/2; (ii) −*x*, *y*−1/2, −*z*+1/2.

### Hydrogen-bond geometry (Å, °)

**Table:**

*D*—H···*A*	*D*—H	H···*A*	*D*···*A*	*D*—H···*A*
C2—H2···O1^iii^	0.94 (2)	2.36 (2)	3.2446 (14)	157.5 (17)

Symmetry codes: (iii) *x*, −*y*+3/2, *z*+1/2.
